# Gases, God and the balance of nature: a commentary on Priestley (1772) ‘Observations on different kinds of air’

**DOI:** 10.1098/rsta.2014.0229

**Published:** 2015-04-13

**Authors:** John G. McEvoy

**Affiliations:** Department of Philosophy, University of Cincinnati, Cincinnati, OH 45221, USA

**Keywords:** air(s), phlogiston, pneumatic chemistry, aerial philosopher

## Abstract

Historians of chemistry usually associate the eighteenth century with the Chemical Revolution, but it could just as readily be called ‘the century of gases’ (or ‘airs’, as they were called in the eighteenth century). In the early part of the century, the British pneumatic chemists struggled to replace the traditional notion ‘Air’, understood as an inert chemical element, with the concept of ‘air’, regarded as the third state of matter, encompassing a wide variety of chemical species. These developments constituted a necessary condition for the Chemical Revolution, which occurred in the latter part of the century. In ‘Observations’, Priestley took pneumatic chemistry to a new level, with the discovery of eight simple inorganic gases. Motivated by his belief in a benevolent God and a pious utilitarianism, Priestly explored the role of the atmosphere in the balance of nature and the politics of the state, which he linked to the movement of Rational Dissent. He styled himself an ‘aerial philosopher’ to signal the interdisciplinary nature of his inquiries, which he regarded not as a branch of ordinary chemistry, but as a mode of thought that encompassed physics, chemistry and natural theology. Priestley saw it as a source of principles and secrets of nature more extensive than that of ‘gravity itself’. This commentary was written to celebrate the 350th anniversary of the journal *Philosophical Transactions of the Royal Society*.

## 1. Introduction

The publication of Joseph Priestley's ‘Observations on different kinds of air’ [1] in the *Philosophical* *Transactions* of 1772 was a seminal moment in our early understanding of the chemistry of gases, or ‘airs’ as they were called in the eighteenth century. Building on the work of the previous generation of British ‘pneumatic chemists’, Priestley provided the first, clear, experimentally articulated statement of ‘air’ as a state, rather than an elemental form, of matter, which like solids and liquids encompasses a variety of chemical species. While his famous rival in the annals of the history of chemistry Antoine Lavoisier selectively incorporated some of the empirical findings of the pneumatic chemists into his revolutionary oxygen theory of chemistry, Priestley integrated them more expansively into a comprehensive ‘doctrine of airs’, which functioned not as a branch of normal chemistry, but as an interdisciplinary mode of inquiry encompassing issues and problems in physics, chemistry, medicine and natural theology. Priestley styled himself an ‘aerial philosopher’, rather than a chemist, not only to register his relative ignorance of traditional chemistry, but also to signal the interdisciplinary nature of his inquiries and their place in a system of thought and practice linked to his important role in the movement of Rational Dissent in eighteenth-century English society.

‘Observations’ covered two distinct domains of inquiry within the newly emergent field of pneumatic chemistry. The paper contained the opening salvo in Priestley's lifelong research programme dedicated to the discovery and examination of different kinds of airs, which eventuated in the discovery of eight new airs. At the same time, he explored the balance in nature between the diminution in volume and vitiation (rendered unfit for respiration) of common air by a variety of processes, including respiration and combustion, and the subsequent restoration of the vitiated air by vegetation and agitation over water. Priestley's inquiries depended on the deployment of new instruments of inquiry, a broad philosophical vision of the unity of God, man and nature, and wider social interests in the utility of science and its role in Enlightenment programmes of individual improvement and social reform.

## 2. Pneumatic chemistry

Chemists at the beginning of the eighteenth century had no clear idea of a gaseous state of matter containing a multiplicity of chemically distinct substances. ‘Pneumatics’, or the physics of gases, emerged during the seventeenth-century scientific revolution as part of the mechanical philosophy of nature. In 1660, Robert Boyle [2, pp. 380–381] used an air pump to show that the mercury column in a barometer tube was supported by the pressure, or expansibility, of the surrounding air. He referred to this property as the ‘elasticity’, or ‘spring’, of the air, which he characterized quantitatively in what became known as Boyle's law of ideal gases (*PV*=const.). He differentiated ‘perennial air’ from vapours and exhalations, which were rendered ‘elastical for a while’ and by ‘manifest outward agents’; Boyle treated elasticity as an essential and immutable property of the microscopic particles of air [3, pp. 97–99].

Boyle's essentialist view of ‘perennial air’ reinforced the widespread seventeenth-century view that air was a simple, elemental and immutable entity, incapable of entering into chemical combinations with other substances [4]. The dependence of chemical phenomena, such as combustion and respiration, on the presence of air was explained in terms of the chemical properties of extraneous particles in the air or in terms of the mechanical motion of the air particles themselves. Similarly, the production of ‘factitious airs’ from a variety of substances was traced to the release of air trapped in the interstices of the substances rather than to the real generation of new air. But Newton [5, pp. 300–302; 6, pp. 395–396] challenged Boyle's essentialist view of air in 1687, when he derived the property of elasticity from a repulsive force that varied inversely as the distance between the particles of air and acted only between nearest neighbours. Newton also argued that at shorter distances, the repulsive force gave way to a short-range attractive force, so that air could exist not only in a ‘free’, or ‘elastic’, state but also, like other substances, in a ‘fix’d', or ‘combined’, state. Newton's aggregative conception of air, which located the property of elasticity between its constituent particles, removed the objection to the idea of air as a chemical entity inherent in Boyle's conception of elasticity as an essential property of the air particles themselves. Newton built the conceptual framework for the experimental work of the British pneumatic chemists.

Stephen Hales [7, pp. 178–180] applied Newtonian principles to the chemical study of air in 1727, when he called upon his fellow ‘Chymists’ to adopt ‘this now fixt, now volatile Proteus among the chymical principles’. Hales measured the amount of air produced by the destructive distillation of a variety of animal, vegetable and mineral substances, but he had no conception of a multiplicity of chemically distinct airs. Joseph Black [8] published the first account of an air different from common air in 1756. The broader interests of Scottish chemists in medical education and economic and social improvement shaped his pneumatic researches [9]. Searching for a milder solvent than ‘caustic alkali’ (calcium hydroxide (Ca(OH)_2_) used by eighteenth-century physicians to dissolve kidney and bladder stones, Black focused his attention on ‘magnesia alba’ (basic magnesium carbonate). He showed that the calcination of magnesium alba resulted in the release of a large quantity of air (carbon dioxide (CO_2_)), which he distinguished from common air by its characteristic odour, greater density, inability to support combustion and respiration, and capacity to precipitate ‘lime-water’ (calcium hydroxide (Ca(OH)_2_) solution). Black used the term ‘fixed air’, which Hales used to denote a physical state of ‘the Air’, to name this new chemical species. In ‘Observations’, Priestley [1, pp. 147–148] adopted Black's usage of ‘fixed air’, distinguishing ‘the other kinds by their properties, or other periphrasis’, without mentioning any ‘hypotheses’ about their internal chemical compositions. Priestley [10, vol. 2, pp. 334–339] used the term ‘air’ to refer to the ‘*mere form* in which a substance is exhibited’, without referencing any of the prevalent views of the underlying constituent cause, such as the ‘elementary fire’, the ‘matter of heat’, or ‘caloric’, of this outward form.

In 1764, the Irish physician David MacBride [11, pp. ix, 272–278; 12] argued that the fixed air released during fermentation and putrefaction was ‘the cement, or bond of union’ of bodies, so that though noxious when breathed ‘ingested fixed air’ offered an appropriate antiseptic, or restorative, in the treatment of ‘the scurvy’, which as a case of putrefaction and decay, was the scourge of the eighteenth-century British navy. But MacBride remained uncertain whether ‘fixed air’ was an ‘originally distinct element in nature’ or a modification of ‘the universal aerial fluid’. It was left to William Brownrigg [13] to make the first unequivocal public statement of the existence of a multiplicity of chemically distinct airs in a paper read to the Royal Society in 1741, but not published until 1765. Brownrigg approached pneumatic chemistry as an Enlightenment physician interested in the medical virtues of spring waters and the deleterious health effects of the vapours, or ‘damps’, encountered in coal mines. He traced the ‘brisk, penetrating, purging’ power of mineral waters to ‘elastic substances’ absorbed during their journey through subterranean passages, but he warned against identifying these vapours with common air ‘in every other respect’ [13, p. 238]. Deploying a simple Newtonian analogy, Brownrigg concluded that ‘two elastic fluids, altho’ they both possess a repulsive quality, may yet in other qualities differ as much as inelastic fluids are found to differ, as water, for example, differs from oil of vitriol' (sulfuric acid (H_2_SO_4_)). Brownrigg identified the conceptual terrain of pneumatic chemistry; Cavendish and especially Priestley delineated its experimental features.

Henry Cavendish [14] clarified the disciplinary boundaries of pneumatic chemistry when he published in the *Philosophical Transactions* of 1766 a paper that dealt with more than one kind of air—fixed air and ‘inflammable air’ (hydrogen (H_2_))—and focused on determining their characteristic properties, rather than their role in a specific chemical reaction or medical procedure. Preparing a sample of fixed air from marble (calcium carbonate (CaCO_3_)) and ‘spirit of salt’ (hydrochloric acid (HCl)), he drew particular attention to its high solubility in water and its capacity to hold in solution the ‘calcareous earths’ and ‘iron impregnations’ that gave to spa and chalybeate waters their medicinal virtues. Priestley's account of fixed air in ‘Observations’ [1, pp. 148–170] expanded on the results of Cavendish and his other predecessors in pneumatic chemists; he also reworked his short pamphlet *Directions for Impregnating Water with Fixed Air* [15], first published in 1772, in which he described a safe, cheap and reliable apparatus for producing carbonated water for the treatment of scurvy and other ‘putrid disorders’. Priestley's intense interest in the utility of science made it ‘one of the happiest thoughts that ever occurred’ to him [10, vol. 2, pp. 269 and 276].

Priestley [1, pp. 170–181] obtained ‘inflammable air’ from metal–acid solutions in the manner described by Cavendish; he also procured less pure samples of the air (probably mixtures of hydrogen and carbon monoxide) by heating other inflammable substances, such as coals, vegetables and ‘animal substances’. He expanded on Cavendish's account of the air's ‘sensible properties’, stressing in particular its peculiar inflammability. Like Cavendish, he obtained from a dilute solution of copper in ‘spirit of salt’ (hydrochloric acid) not inflammable air as expected, but the highly soluble ‘marine acid air’ (anhydrous hydrogen chloride (HCl)), which he collected over ‘quicksilver’ (mercury) instead of water [1, pp. 234–244]. Repeating Cavendish's experiments on metals—except lead and tin—dissolved in ‘spirit of nitre’ (nitric acid (HNO_3_)), Priestley obtained ‘nitrous air’ (nitric oxide (NO)), which played a prominent role in his subsequent pneumatic inquiries [1, pp. 210–224].

In recognition of his earlier work in electricity, optics, the history of science and pneumatic chemistry, the Royal Society awarded Priestley the prestigious Copley Medal in 1773. Published in the same year, ‘Observations’ marked an intensification of his interests in pneumatic chemistry, which between 1774 and 1790 resulted in the publication of six volumes of *Experiments and Observations on Different Kinds of Air* [10,16,17], as well as a dozen articles in the *Philosophical Transactions*. In these publications, Priestley provided the scientific community with detailed accounts of the preparation, collection and manipulation of a total of eight new airs, along with those covered in ‘Observations’ [18,19, pp. 145–170]. The new airs were: ‘marine acid air’; ‘inflammable nitrous air’ (nitrous oxide (N_2_O)); ‘red nitrous vapour’ (nitrogen dioxide (N_2_O_4_)); ‘alkaline air’ (ammonia (NH_3_)); ‘vitriolic acid air’ (sulfur dioxide (SO_2_)); ‘fluor acid air’ (silicon tetrafluoride (SiF_4_)); ‘heavy inflammable air’ (carbon monoxide (CO)); and most famous of all, ‘dephlogisticated air’ (oxygen (O_2_)).

**Figure 1.**
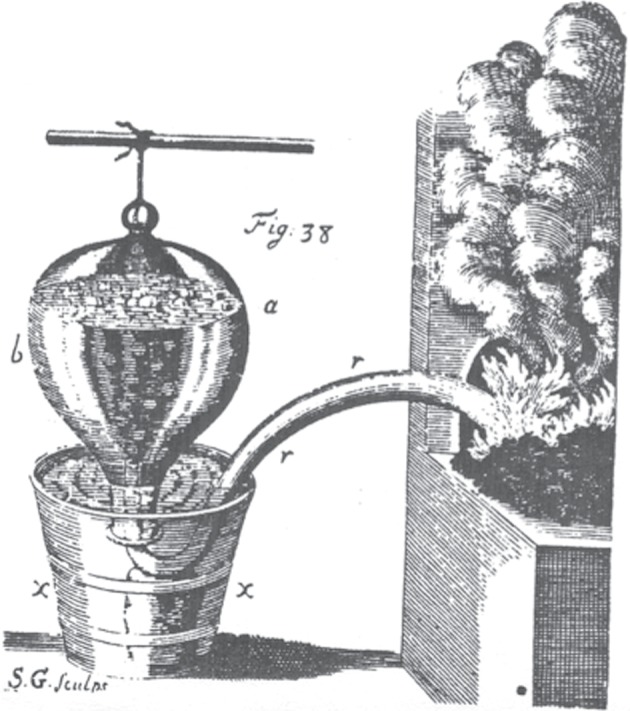
Hales's apparatus from *Vegetable Statics* (London 1727). Copyright The Royal Society.

**Figure 2.**
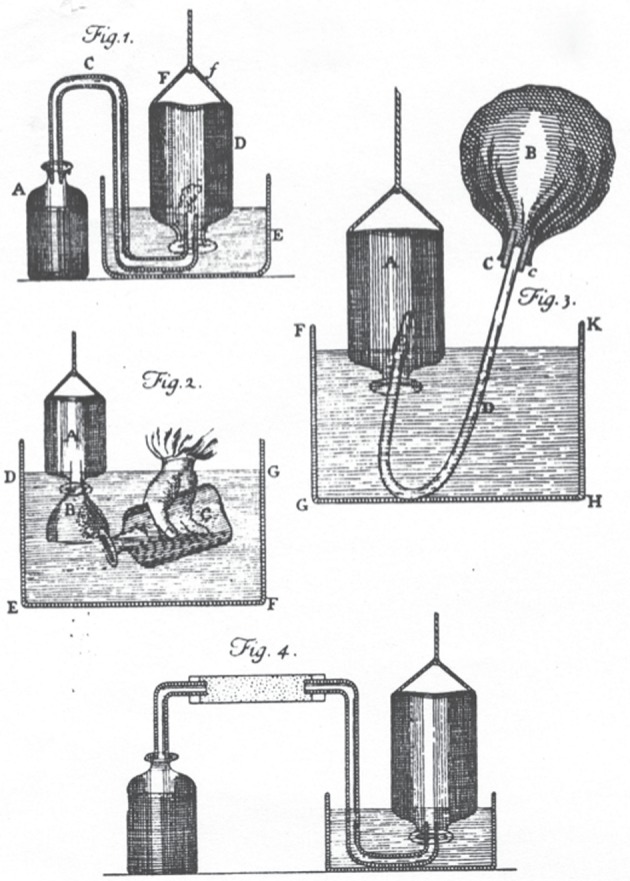
Cavendish's apparatus from *Philosophical Transactions* (1766). Copyright The Royal Society.

**Figure 3.**
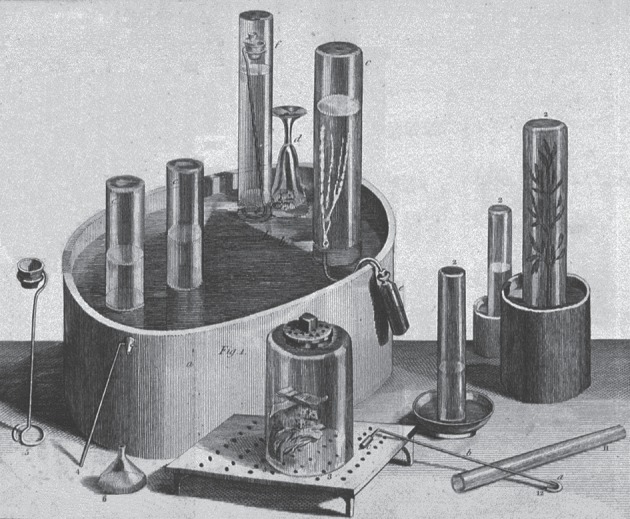
Priestley's apparatus from *Experiments and Observations on Different Kinds of Air* (London 1774). Copyright The Royal Society.

Priestley's experimental success resulted mainly from his ability to design simple apparatus and his skill in manipulating them [20,21]. Hales invented the ‘pneumatic trough’ as a way of using the upward displacement of water to isolate and collect such an insensible and rarified substance as ‘the Air’, but lacking the concept of a multiplicity of chemically distinct airs, he focused solely on measuring the amount of ‘Air’ produced by a chemical substance or reaction and developed no techniques for its manipulation (figure 1). It was left to Cavendish, with his growing awareness of a plurality of airs, to show how to manipulate them under water (figure 2). Priestley's clearer sense of the endless multiplicity of chemically distinct airs is evident in the multiplicity and variety of the apparatus he used for their production, isolation and manipulation (figure 3). Like Hales, Brownrigg and Cavendish, Priestley relied mainly on readily available objects, such as kitchen jars, wooden or earthen troughs, gun barrels and clay pipe stems. The use of glass vessels to better contain and monitor airs, by the progressive displacement of water, was another important development, as was the substitution of ‘quicksilver’ for water in the collection and manipulation of airs soluble in water. Even when he used more specialized apparatus and techniques, Priestley always insisted on the simplicity and easy availability of his apparatus and instruments. Priestley's valorization of ease and simplicity in the laboratory was more than a matter of preferred technique; it carried with it important epistemological and metaphysical interests that transcended the disciplinary boundaries of pneumatic chemistry.

## 3. Aerial philosopher

As an ‘aerial philosopher’ [22], Priestley claimed that pneumatic chemistry was ‘not now a business of air only’, but was about ‘principles of more extensive influence than even that of gravity’ [10, p. viii; 18, Part III, pp. 156–157]. These sentiments were paramount in Priestley's detailed examination of the diminution in volume and vitiation (rendered unfit for respiration) of common air by such processes as combustion, calcination, respiration, putrefaction, mixture with nitrous air and other ‘noxious processes’, and the provisions in nature, such as vegetation and the agitation of seas and lakes, for the subsequent restoration and improvement of the vitiated air [1, pp. 162–170, 181–209, 225–234]. Priestley's firm belief in nature as a deterministic system of benevolence, created by God to generate good out of evil, provided the general conceptual framework, and influenced some of the experimental aspects, of this line of inquiry [18, Part II, pp. 96–103; 18, Part III, pp. 158–164]. While his results laid the groundwork for the important photosynthesis experiments performed by Jan Ingenhousz and Jean Senebier, they also provided Priestley with evidence of the overriding importance of natural philosophy in human affairs. Under the sway of David Hartley's associationist psychology, according to which the development of the mind is determined by the mechanical law of the association of ideas, Priestley maintained that the perfectibility of the individual was the inevitable consequence of an increasingly adequate knowledge of a benevolent natural world. While natural philosophy inculcated sentiments of benevolence and piety in its practitioners, these sentiments, in turn, facilitated the greater comprehension of nature. Priestley's theism placed great store in natural philosophy, further integrating it into a wider matrix of thought and practice rooted in his life of Rational Dissent [23,24].

Priestley was born in 1733 in the Calvinist stronghold of the West Riding of Yorkshire, but by the time he graduated from the Dissenting Academy at Daventry in 1755, he had abandoned the Calvinist doctrines of original sin and atonement in favour of the rational principles of Unitarianism, which rejected the Trinity and upheld the perfectibility of man. He joined the faculty at Warrington Academy in 1761, where he developed a philosophy of science which based the pursuit of knowledge on the sensory experience and the mechanical association of ideas of individual inquirers, unencumbered by prejudice and received opinion; he also linked Bacon's vision of the improvement of society through the practical benefits of a science-based commerce to the entrepreneurial activities and commercial interests of English Dissenters. While in Leeds and then Calne, Wiltshire, he deployed the doctrine of pious utilitarianism to champion, as part of God's providential plan for the progressive amelioration of man and nature, the national struggle of Dissenters for greater civil, political and economic liberties. Priestley's progressive utilitarian ethos came to the fore when he moved to Birmingham in 1780 and joined the Lunar Society, an elite group of local gentlemen, Dissenters and industrialists who applied science to the solution of the problems of eighteenth-century life [25,26].

Priestley's pious utilitarianism is discernible in the enthusiasm with which he pursued his project for the use of carbonated water in the treatment of scurvy; this project was also a response to a broader problem in Enlightenment medicine, which looked to science to curb the diseases (fevers) of modern institutional populations. Although the Admiralty decided in the early 1790s to use lemon juice to combat scurvy, a version of Priestley's apparatus, called the ‘gasogene’, became a popular commodity in the burgeoning eighteenth-century market for consumer goods and medical therapies [24, pp. 105–128]. Priestley's ‘eudiometer’, which used the measured diminution in volume of a mixture of common air and nitrous air to determine the purity of the air, also became an important tool in Enlightenment movements, both in Britain and on the Continent, for sanitary reform, urban planning and social control through the maintenance and improvement of the health of human individuals and populations [27, pp. 292–314]. But eudiometric practitioners failed to recognize standardized procedures and obtain uniform results; it was almost completely abandoned in the 1780s when its practitioners conceded that the nitrous air test did not reveal all the bad qualities of an air, only its degree of phlogistication (or lack of oxygen) [24, pp. 117–128; 27, pp. 306–318]. Enlightenment pneumatic medicine reached its culmination in Britain in the Pneumatic Institution, founded by Thomas Beddoes in Bristol in 1798 in order to explore the effects of airs other than common air on a variety of familiar ailments; but the hilarious goings on with nitrous air (laughing gas) played into the hands of conservative forces, including Edmund Burke and the editors of the Anti-Jacobin Review, who viewed the Enlightenment as a period of follies, illusions and vain enthusiasms which needed to be brought to a speedy end. The conservative backlash ruined Beddoes's reputation and eventually drove Priestley into lonely exile in Northumberland, Pennsylvania [24, pp. 263–401].

Priestley's broad philosophical vision issued in a powerful set of epistemological principles and methodological guidelines which regulated his pneumatic inquiries according to a strict distinction between ‘facts’ and ‘hypotheses’ [23, pp. 399–404; 18, Part 1, pp. 31–36]. The injunction to police the boundaries between facts and hypotheses is immediately evident in Priestley's view of the phlogiston theory as a ‘mere hypothesis’, which like all hypotheses was necessary for the discovery of ‘new facts’, but bereft of any lasting explanatory worth [28]. Priestley modelled the version of phlogiston he used in ‘Observations’ on Cavendish's appeal to the standard Stahlian notion of a generic ‘principle of inflammability’ (present in all combustible substances, including metals) to explain the production of inflammable air from dilute metal–acid solutions [18, Part 1, pp. 43–45, Part II, pp. 104–105]. But Priestley soon loosened phlogiston from its tradition identification with the principle of inflammability, turning it into a veritable Proteus of cosmic proportion 18, Part III, pp. 158–164; 29]. He developed a phlogistic account of the role of respiration, which emitted phlogiston, and vegetation, which reabsorbed it, in the balanced economy of nature. Towards the end of his life, he detected a ‘regular graduation’, from most pure to least pure, of dephlogisticated air, common air, phlogisticated air and nitrous air, and suggested ‘all these kinds of air differ chiefly in the quantity of *phlogiston* they contain’ [19, p. 179]. Whatever status or function Priestley ascribed to phlogiston, he never lost sight of its hypothetical nature and its inferior epistemological status vis-à-vis the facts it unearthed.

Priestley's associationist view of the passivity of the knowing mind, bereft of preconceived opinion and attuned to the deterministic structure of nature, established ‘facts’, or congeries of ‘sensible properties’, as completely determined by external reality [23, pp. 348–357, 374–380]. Hypotheses about the underlying causes—such as chemical compositions or reaction mechanisms—of these sensible properties and their modifications threatened, if not clearly distinguished from the facts of the matter, to sully objective knowledge with mere opinion, subtle bias or outright dogma. For Priestley, the fundamental business of the natural philosopher, and the bedrock of his pneumatic inquiries, consisted of the heuristic deployment of hypotheses in the proliferation and accumulation of enough facts to allow, at some future date, the induction of a ‘general theory’ to which they are reduced [10, vol. 2, p. vii; 18, Part III, pp. 153–157].

The distinction tween facts and hypotheses influenced the narrative style of ‘Observations’. Priestley [30, pp. ii–xx, 576–577; 18, Part 1, pp. 36–39] developed the ‘analytical and historical style’ of writing in his earlier work in electricity and its history, for which he was elected a member of the Royal Society in 1766, and he continued to adopt it in all his subsequent scientific publications. He designed his new style to offset the prevalent ‘synthetic style’ of presentation, which by assimilating science to the axiomatic theoretical activity of a few men of genius, such as Isaac Newton, discouraged many people from making discoveries of their own. Priestley wrote for a scientific community envisioned by his Dissenting interests in individual liberty, associationist notions of the epistemological equality of all men, and Baconian principles of the division of labour [31, pp. 132–135]. According to Priestley, discoveries in natural philosophy depended not on individual moments of illumination in men of genius, but on the ability to take infinite pains in gathering facts, and could therefore be made by anyone. Designed to facilitate the inquiries of contemporary and future experimentalists, Priestley's scientific publications bristled with observations and experimental details, without much concern for the formal niceties of his theoretical constructions and hypothetical explanations. These sentiments induced Priestley to include in ‘Observations’ [1, pp. 253–256, 257–264] and his other major scientific publications, separate sections dealing with ‘Miscellaneous Observations’ and ‘Appendices’ consisting of notes and letters from members of a growing community of practitioners, or ‘Queries, Speculations and Hints’ for others to pursue [10, vol. 1, pp. 258–285; 18, Part II, pp. 109–112]. The importance Priestley placed on policing the boundaries between ‘facts’ and ‘hypotheses’ is evident in the penultimate section of the fifth volume of *Experiments and Observation on Different Kinds of Air* [16, vol. 2, pp. 325–365, published in 1781], where he took the unusual step of providing a 40-page ‘Summary View of All the most remarkable Facts in this and the four preceding volumes’. Priestley here compiled the more significant and agreed-upon ‘general propositions’ concerning the sensory properties and modifications of various airs (and other relevant substances), while carefully avoiding any mention of ‘hypotheses’ concerning their internal chemical composition and underlying reaction mechanisms responsible for these properties and their transformation.

## 4. The chemical revolution and the ‘doctrine and airs’

Priestley's lasting reputation in science rests, in large part, on his discovery in August 1775 of an air, by heating *mercurius calcinatus per se* (mercuric oxide (HgO)), in which a candle burned brighter and a mouse lived longer than in common air [10, vol. 2, pp. 33–101, vol. 3, pp. xxii–xxxi, 41–45; 18, Part III, pp. 164–175]. While Priestley called this air ‘dephlogisticated air’, Lavoisier identified it as ‘oxygen’, composed of the ‘acidifying principle’ and ‘caloric’, which he used to revolutionize chemistry [29]. Priestley was uncompromising in his opposition to the ‘French system’ of chemistry, detecting in its attempt to impose theoretical unity on the chemical community a threat to the individual experimenter that paralleled the usurped authority of the eighteenth-century religious state [31, pp. 131–142]. These concerns were uppermost in Priestley's mind in the 1780s and 1790s, as the political struggle of the Dissenters to repeal the Test and Corporation Acts intensified. The English press and government declared Priestley's support of the American and French Revolutions ‘seditious’, and on 14 July 1789, the ‘Church-and-King mob’ destroyed his house and laboratory. In 1794, Priestley eventually fled to the United States, where he regarded it as his liberal and Enlightenment duty to oppose the ‘new system of chemistry to his dying day’, which occurred on 6 February 1804 in Northumberland, Pennsylvania [26, pp. 262–401].

Contrary to the traditional view of Priestley's role in the Chemical Revolution, his main focus was on the critical evaluation of the oxygen theory rather than the speculative defence of the phlogiston theory, though he did believe that, in the current state of chemistry, rejection of the former entailed acceptance of the latter [28,32]. He showed that the phlogiston hypothesis, which upheld the traditional view of elemental water, was *not* clearly evidentially inferior to Lavoisier's oxygen theory and its revolutionary view of the compound nature of water [33, pp. 1–70]. He also expressed a general, epistemological scepticism about Lavoisier's results; he cited—as confusing the phenomenological description of facts with a hypothetical interpretation of them—the French chemist's excessive use of sophisticated experimental procedures, elaborate and expensive laboratory apparatus, and overly complex ‘computation’ and ‘allowance’ to generate the idealized data necessary for the formulation of ‘true equations’ [31, p. 132]. Although Priestley opted for the ‘phlogistic hypothesis’, he recognized that it was ‘not without its difficulties’, but he insisted that it shared these difficulties with Lavoisier's theory. Whether in the form of phlogiston or caloric, each theory violated a shared epistemic commitment to a chemistry of ponderable substances, isolable in the laboratory [28, pp. 203–205]. Priestley valued both hypotheses as heuristic devices for the generation of new facts in a patient, humble experimental approach to God's infinite creation.

Priestley and other phlogistic chemists faulted Lavoisier not for emphasizing the importance of quantitative analysis, but for narrowing the domain of chemistry to the stoichiometric determination of the relative quantities of reactants and products in chemical reactions [19, pp. 207–236]. Lavoisier's conception of the aerial domain was also considerably narrower than Priestley's doctrine of airs. Lavoisier's understanding of the chemical nature of air was shaped by the previous generation of French Stahlians, who worked within Hales's problematic of the chemical identity of elemental air. Even when he arrived, somewhat belatedly, in the early 1780s at a clear conception of the multiplicity of chemically distinct airs, the unitary logic of elemental air survived in his view of oxygen as the universal principle of acidity, the active ingredient in all chemical reactions [19, pp. 51–62].

Lavoisier worked within narrower disciplinary constraints than Priestley. Whereas the French Stahlians represented an activity of successive generations of chemists with a distinct disciplinary identity, the British pneumatic chemists and their results were not seen as more particularly belonging to chemistry than to physics, medicine or natural theology [34,35]. Indeed, Priestley envisioned his doctrine of airs as more powerful and fundamental than ordinary chemistry; he even ventured the idea of chemistry conducted entirely in the ‘aerial state’. Critical of Lavoisier's elaborate laboratory instruments and complex procedures, he declared in 1790 that ‘by working in a tub of water, or a bason [sic] of quicksilver, we may perhaps discover principles of even more extensive influence of event that of gravity’ [17, p. xxiv]. Priestley's keen anticipation of the endless ‘future progress’ of his doctrine of airs registered a consilience of considerations, including the de facto progress of his own inquiries, his belief that as rarefied distillates of solids and liquids, airs brought us closer to the real natures and affinities of things, and his abiding sense of the role of natural philosophy in the endless progress and perfectibility of human nature through the comprehension of an infinite world, bristling with ‘novelty’ and grounded in God's benevolent fecundity [10, vol. 3, p. ix].

Priestley's vision did not appeal to his contemporaries. The work of John Dalton and Joseph Louis Gay-Lussac on the law of combining volumes, in the early nineteenth century, made pneumatic chemistry an unremarkable branch of normal chemistry. Priestley's doctrine of airs entered modern chemistry in this prosaic guise, but in order to understand its global significance and real impact on his contemporaries, especially Lavoisier, the historian of chemistry must understand it in its eighteenth-century interdisciplinary grandeur, seeking principles in nature of more extensive influence than ‘that of gravity itself’. An adequate account of Priestley's doctrine of airs and his role in the Chemical Revolution must do justice to his self-proclaimed image as a ‘comet in the system’, offsetting the stifling tendencies of disciplinary uniformity and conformity with a plethora of new discoveries and a mode of theory and practice based on a synoptic sense of man's unfolding comprehension of nature.

## Author profile

**John McEvoy**, as professor of philosophy at the University of Cincinnati, teaches and researches a variety of topics encompassed by the domains of the history and philosophy of science, science studies and aspects of the Marxian corpus that relate to these interests. This interdisciplinary activity has drawn him into a nexus of problems concerning the relation of the history of science to its historiography. Armed in the early 1960s with an honours (2.1) degree in chemistry (Liverpool) and a postgraduate Certificate in the History and Philosophy of Science (Cambridge), he was at a loss for an appropriate dissertation topic when the late historian of chemistry Satish Kapoor pointed him in the direction of Joseph Priestley and the library of the British Museum where most of the relevant documents were housed. He immersed himself in this treasure trove of information, which provided the foundation of a PhD dissertation at the University of Pittsburgh and much of his subsequent research.
